# Crohn's Disease Discovery to Modern Treatment: A Gastroenterology Fellow's Experience at Mount Sinai With the Crohn's & Colitis Foundation Visiting Fellowship Program

**DOI:** 10.14309/crj.0000000000001533

**Published:** 2024-10-10

**Authors:** Robert James Pattison, Arafa Djalal

**Affiliations:** 1 Department of Gastroenterology, Straub Medical Center, Honolulu, HI; 2 Mt. Sinai Medical Center, New York, NY

In 1932, Dr. Burill Crohn, MD, first described Crohn's disease in a series of patients with inflammation in the terminal ileum at the Mount Sinai Hospital in New York City.^1^ Today, a patient with inflammatory bowel disease (IBD) has more medical and surgical options than ever before. Even with the treatments available today, up to 80% of patients fail to reach remission.^2–4^ Patients face the uncertainty of a life-long disease whose pathogenesis has yet to be clearly understood. Nevertheless, the incidence and prevalence of IBD continue to grow worldwide. With this increasing patient volume, there is an increasing need for gastroenterologists who are capable of providing optimal care for these patients. To meet this need, non–ACGME-accredited advanced fellowships in IBD are offered at referral centers around the country.^5^ The Crohn's & Colitis Foundation Visiting IBD Fellowship Program was established to train gastroenterology fellows on IBD care at leading IBD centers of excellence. The purpose of this editorial is to tell you about my experience as a visiting fellow in this program.

In 2006, the Crohn's & Colitis Foundation started a program called the Visiting IBD Fellowship Program. Since that time, they have sponsored visiting fellowships for nearly 300 fellows in the United States. The program offers financial support covering most expenses for a fellow to work at a leading IBD center of excellence. The program aims to help gastroenterology fellows gain exposure to patients with IBD at a high-volume referral center. Pediatric and adult gastroenterology fellows are eligible to apply to the program in their second or third year of fellowship.

My visiting fellowship took place at The Susan and Leonard Feinstein IBD Clinical Center at Mount Sinai Hospital in New York City. This is a state-of-the-art comprehensive care center for patients of all ages living with IBD. The center incorporates unique programs involving services for all aspects of IBD care. The concept uses a medical home model for the patient with IBD. Within this medical home, they offer psychological services aimed at building resilience in a program called Gaining Resilience Through Transitions (GRITT-IBD). They have services for females with IBD who want to conceive in a program called Preconception and Pregnancy Planning. There is also an abundance of ongoing clinical trials in which patients can be enrolled. The clinic offers simultaneous appointments with other specialists such as rheumatology in the IBDs and Arthritis Clinic for patients also followed by both services. Practically, this means the patient is able to see both specialists at the same appointment—increasing coordination of care and patient satisfaction. There are attendings who subspecialize in various areas of IBD with respect to the extraintestinal manifestations of the disease such as perianal disease, arthritis, pouchitis, and fertility. They are one of 3 programs in the United States to offer a point-of-care intestinal ultrasound program for real-time monitoring of disease activity. As a visiting fellow, I rotated with each individual provider within each specialty area of the clinic. The center is directed by 2 of the most impactful researchers in the field: Jean-Frederic Colombel, MD, and Marla Dubinsky, MD, with the Gastroenterology Division led by Bruce Sands, MD. The 1-year Advanced IBD Fellowship is directed by James Marion, MD, who also offers chromoendoscopy.

In my time at Mount Sinai, I saw more than 350 patients living with IBD from every walk of life. Their patient population is in every sense of the word diverse. The patients range from Wall Street Executives to individuals coming from abroad for another opinion to homeless people. They range from patients who have had multiple bowel resections and are on advanced medications to those whose disease has been held in check for years with mesalamine. There was one constant in every interaction: Each patient is treated with the utmost care and attention. They tailor therapy to the patient's disease phenotype and needs. They have employees advocating and fighting for patients whether that be with an insurance company or attendings taking time to call the patient to make sure they stay on infusion schedules. In each patient interaction, I witnessed an attitude of care and sincerity in trying to get the patient better. I observed the intangibles in the spirit of the staff. Internationally renowned attendings and researchers in their field generously make themselves available to patients with MyChart and even sharing personal phone numbers to allow rapid follow-up in complex cases. My observation was that there was nothing the team at Mount Sinai would not do to get their patients well.

My schedule was set up with a clinic from 8 am to 5 pm, typically working with a different clinician in each half day within a different subspecialty of IBD. There were dedicated IBD conferences weekly and morning report sessions. Other days were split up between the various collaborative clinics such as the IBD who want to conceive in a program called Preconception and Pregnancy Planning, IBDs and Arthritis Clinic in conjunction with rheumatology or clinics where patients were seen in collaboration with IBD focused general or colorectal surgeons. Some patients would come specifically for the other resources the IBD home offers specifically social workers, psychologists, and dieticians who all tailor their services toward improving the lives of patients suffering from IBD. In providing these services the patient care is tailored and individualized.

For many patients whose lives have been impacted by their disease, the IBD Center at Mount Sinai represents a final hope. The trust that the patients give the IBD team is met with the tireless effort of each provider and staff member to care for each patient in an all-encompassing way. Their example was inspiring and something that will forever serve as a model of excellence for me.

The Crohn's & Colitis Foundation Visiting IBD Fellowship Program offers a unique exposure to referral-based patients with IBD and the multidisciplinary team it takes to manage them. The opportunity to work with the leaders in the field and see their dedication to their patients was truly inspiring and one of the most rewarding experiences of my educational journey. I highly recommend any gastroenterology fellow who has an interest in IBD consider applying to the Crohn's & Colitis Foundation Visiting IBD Fellowship Program.

## DISCLOSURES

Author contributions: RJ Pattison: writing, editing, submitting. A. Djalal: editing.

Financial disclosure: None to report.
